# Violence Against Healthcare Workers in a University Hospital of Central Italy: How Risk Management Interventions Can Help Change a Trend

**DOI:** 10.3390/healthcare13040409

**Published:** 2025-02-14

**Authors:** Lavinia Bianco, Stefania Oliva, Fabiano Grassi, Jan Francesco Arena, Mariarosaria Aromatario, Stefano Ferracuti, Simona Abate, Christian Napoli, Antonio Del Casale

**Affiliations:** 1 Department of Public Health and Infectious Diseases, “Sapienza” University of Rome, 00185 Rome, Italy; stefania.oliva@uniroma1.it (S.O.); fabiano.grassi@uniroma1.it (F.G.); 2 National Institute for Health, Migration and Poverty (NIHMP), 00153 Rome, Italy; mariarosaria.aromatario@inmp.it; 3 Department of Dynamic and Clinical Psychology and Health Studies, “Sapienza” University of Rome, 00185 Rome, Italy; janfrancesco.arena@uniroma1.it (J.F.A.); antonio.delcasale@uniroma1.it (A.D.C.); 4 Department of Human Neurosciences, “Sapienza” University of Rome, 00185 Rome, Italy; stefano.ferracuti@uniroma1.it; 5 Sant’Andrea Hospital, “Sapienza” University of Rome, 00189 Rome, Italy; simona.abate@uniroma1.it; 6 Department of Medical Surgical Sciences and Translational Medicine, “Sapienza” University of Rome, 00189 Rome, Italy

**Keywords:** workplace violence, healthcare workers, violence, aggression, risk management, root cause analysis, preventive measures, psychological support, hospital management

## Abstract

**Background/Objectives**: Violence against workers (physical, verbal, or psychological abuse), even if it affects all professional categories, is up to ten times higher in healthcare workers. A University Hospital of Central Italy has gradually implemented a series of preventive measures, which might have impacted the trend of the phenomenon. **Methods**: In order to monitor the episodes, an aggressive event reporting form was adopted throughout the hospital. Data extracted both from this form and the root cause analysis documentation regarding the period January 2019–December 2023 were used. Descriptive statistical analysis was performed using the Chi-squares test, while the join point regression program was used to analyze the trends. **Results**: The average age of the sample is 43.5 years, with twice as many female workers as male workers. Nurses are more frequently involved (76.6%), and 58% of the events involved 2 to 4 HWs. Verbal violence was reported by 51.2% of HWs, and over 35% of them did not suffer any damage, while 25% suffered mild to moderate damage (illness, injury, or material harm, whether physical or psychological). The hospital divisions which are primarily concerned are the Psychiatry department (36.2%) and the Emergency Room (33.4%). There is no difference in hospital management of aggressive events between males and females. Join point regression analyses showed that there was a significant increase in the reporting of episodes of aggression. **Conclusions**: The presence of a strong culture of reporting among HWs guarantees a seemingly constant increase in the reporting of nonphysical forms of violence. The statistically significant differences found will allow hospital management to categorize the risk levels and act accordingly.

## 1. Introduction

In recent years, violence against workers has become a significant public health issue. The international literature provides a number of meanings for the term “workplace violence”, including one from the National Institute for Occupational Safety and Health (NIOSH), which identifies it as “any physical assault or attempted assault, threatening behavior, or verbal abuse that occurs in the workplace” [1]. According to the joint definition by the World Health Organization (WHO) and the International Labour Organization (ILO), workplace violence is defined as “all incidents in which workers are abused, threatened, or assaulted in work-related situations, including commuting, that involve an implicit or explicit risk to their safety, well-being, or health” [2]. Thus, when talking about violence against workers, one must refer to any form of aggression, be it physical, verbal, or psychological abuse, including threats, harassment, physical or sexual assault, racial harassment, cyber persecution, or bullying [3,4].

This phenomenon affects all professional categories globally. However, healthcare workers are the most affected: compared to other work areas, events in this field occur far more frequently, with exposure up to ten times higher than that of workers in other sectors [5]. For instance, the WHO reports that up to 38% of healthcare workers face scenarios of brutality at least once during their career (and they face them 16 times more than in other professional fields), while the CDC (Centers for Disease Control and Prevention) states that 70% of private industry workers affected by workplace violence belonged to the healthcare and/or social assistance field [4].

A recent umbrella systematic review and meta-analysis, despite taking into account the variability of certain data points, showed that the prevalence of violent acts experienced by healthcare workers in studies is 78.9% [3].

Studies conducted in Italy also confirm the international trend of increased violence against healthcare workers. Specifically, according to one study, the percentage of nurses reporting workplace violence incidents of any nature ranges from 48.6% to 65.9% [6]. This percentage rises to 76% regarding verbal violence experienced by Emergency Room nurses [7], whereas another study on the risks and effects of workplace violence on nurses and doctors in emergency and urgent care areas confirmed the global findings [8]. Even if major international organizations such as the WHO and ILO indicate that up to 50% of workers might be affected by this phenomenon [2], it appears to be globally underestimated due to various factors, including a lack of effective reporting systems and a tendency to view such incidents as an inherent aspect of the job [3,9]. Consequently, the propensity to not disclose violence episodes is a fairly common phenomenon, especially in some contexts, with some studies estimating that 70–80% of incidents go unreported [10,11].

The significance of the issue as a public health problem and the alarming increase in the phenomenon are both highlighted by the expanding number of scientific papers published globally, as well as the media’s attention to increasingly regular instances [12]. These studies focus on measuring and analyzing aggressive events and their impact on workers’ health [13,14,15] and on local and global prevention and mitigation interventions; the latter include actions such as issuing specific guidelines and regulations and developing validated and shared international tools [3,16,17]. The high number of scientific works on the topic is due to the fact that such events have an impact on working conditions and the quality of healthcare safety, as well as reflecting a specific professional risk that requires adequate preventive measures. Indeed, a violent episode can have a severe impact on workers, including extensive injuries, death, psychological distress, burnout, higher rate of medical errors, worse patient outcomes, and increased attrition [4].

From this perspective, several measures have been adopted over the years to mitigate the phenomenon in Italy. The first one is the Health Ministry Recommendation No. 8 (November 2007), “Preventing acts of violence against healthcare workers”, which emphasizes that, to prevent acts of violence, every healthcare organization must identify risk factors for staff safety and implement the most appropriate strategies [2]. Specifically, the Health Ministry Recommendation, according to the international literature, highlights the following actions:

- Developing a prevention program;
- Analyzing work circumstances as well as innate and modifiable risk factors;
- Training and raising awareness among staff;
- Promoting a culture of reporting [18].

Collaborations with law enforcement or other organizations capable of providing valid support are also advised, as are any structural and organizational changes meant to lower the risk.

Afterwards, in August 2020, Law No. 113 “Provisions on safety for healthcare and social health professionals in the exercise of their functions” was approved by the Italian Parliament: it imposes harsher penalties for acts of violence against healthcare workers, especially in cases of personal injury [19]. Furthermore, article 2 of the same law established the National Observatory on the Safety of Healthcare and Social Health Professionals (ONSEPS), which has the specific tasks of monitoring, studying, and promoting initiatives to ensure professionals’ safety [19]. The fight against violence episodes was further strengthened by Law No. 31 of 2023, recently enacted, which allows for automatic protection even in cases of minor injuries, regardless of the victim’s willingness to file a complaint [20].

According to these laws and to the Health Ministry Recommendation, a University Hospital of Central Italy has gradually implemented a series of preventive measures. Firstly, since 2018, a culture of reporting has been promoted to raise awareness among healthcare workers and to carry out training activities. Each report is followed by appropriate actions by the Risk Management Unit (RMU), the Prevention and Protection Service Manager, and a representative of the Psychological Care Unit. These actions are part of a hospital procedure of root cause analysis (clinical audits, incident reporting, and adverse event reporting) which includes using the Lazio region reporting system, along with the related IT flow, to fuel the adverse events national database. The classification of the damage, which, in the context of workplace safety, is intended as “any form of illness, injury, or material harm, whether physical or psychological that a worker may experience from accidents related to their place of employment”, must be given special consideration when populating said database [21].

Concurrently and over the last few years, RMU has implemented procedures and operational protocols to reduce the likelihood of incidents and facilitate their management, especially in high-risk settings such as the Emergency Department (ED) and Psychiatry ward. Regarding the ED, the presence of law enforcement has been increased to deter aggressive behavior; the ED premises were renovated to obtain greater efficiency in the management of low-priority tags, improve the triage working condition, and provide timely information on the status of patients’ health to family members, thus also limiting episodes of violence against healthcare workers. Lastly, nursing and medical staff received training, and a random monitoring system of the ambulance block in the Emergency Room was implemented [22].

The aim of this study was to examine the incidence of the phenomenon from 2019 to 2023 in a University Hospital of Central Italy in order to determine whether and to what extent the measures adopted in compliance with this new legislative background have had an impact on it.

## 2. Materials and Methods

### 2.1. Monitoring Process

Since the beginning, the monitoring of aggressive acts has been initiated through the reporting system by the assaulted operator; however, as previously noted, this activity was initially hindered by operators’ tendency not to report, and only in recent years, after appropriate training, has a higher reporting frequency been observed. Regarding this monitoring activity, RMU utilizes an aggressive event reporting form created by Regione Lazio’s Regional Center of Clinical Risk (Centro Regionale Rischio Clinico—CRRC) [23] and adopted through hospital procedure. This form contains the following information:

- Personal information of the healthcare worker (name, surname, date of birth, care unit);
- Characteristics of the aggressive episode (single or multi-operator, location, work schedule, care setting);
- Characteristics related to the aggressor (patient, family member, visitor, other);
- Type of aggression suffered (verbal, physical, or both);
- Factors favoring the episode and factors that could have mitigated the risk associated with the event;
- Type and severity of the damage suffered (physical or psychological, certified or not);
- Actions following the event (injury, report of the aggressor).

Additionally, the form provides space for a brief description of the event. For an event involving multiple operators, the form allows each operator to complete it with the relevant information (care unit, type of aggression, and damage potentially suffered). This form, available to all care units of the hospital, is the tool used by operators for reporting and, at the same time, it is used by the Risk Management Unit for monitoring the reported events, which, depending on the outcome, are classified as “adverse events” (from no damage to moderate damage) or as “sentinel events” (from severe damage to death).

### 2.2. Data Extraction and Analysis

For the purposes of this study, data extracted from the monitoring activities carried out by the Risk Management Unit (RMU) of a University Hospital of Central Italy during the timeframe January 2019 through December 2023 were used. All data contained in both the reporting form and on the root cause analysis documentation, including those belonging to the Prevention and Protection Service and to the Psychological Care Unit, were collected in an appropriate database created with the use of Excel (Microsoft Office, 2019).

Descriptive and inferential univariate statistical analysis was performed using the Chi-squared test and Fisher’s exact test for the independence of categorical variables, whereas the T-test (heteroskedastic with two tails) was used for quantitative variables (e.g., age). A *p*-value < 0.05 was assumed as the significance level.

To analyze trends, we performed a regression using the join point regression program (version 5.2.0.0, 2024). We analyzed the quarterly percent change (QPC) from April 2019 to December 2023. We used the quarterly episodes of aggression rate as the dependent variable, assuming constant variance (homoscedasticity) and logarithmic transformation. We set a maximum number of 3 join points and used a permutation test with an overall significance level set to *p* < 0.05.

## 3. Results

### 3.1. Descriptive and Inferential Statistics

The characteristics of the 209 healthcare workers involved in the reported violent acts are shown in Table 1. The average age of the sample is 43.5 years, and there are approximately twice as many female workers as male workers. Nurses are the most frequently involved professional category (76.6%). Concerning the total number of HWs involved, 51.2% of them reported experiencing verbal violence, while 30.1% experienced both verbal and physical aggression. The difference between males and females regarding both age and the type of aggression experienced was found to be statistically significant (*p* < 0.05). The aggressive episodes took place in descending order during the morning, afternoon, and night shifts, with 71.8% of healthcare workers being attacked by a patient. Over 35% of the healthcare workers involved did not suffer any damage, while 25% suffered mild to moderate damage.

The characteristics of the aggressive events are shown in Table 2, both in their total number and divided by work shifts (morning, afternoon, night). Although the total number of aggressors is 131, the number of aggressive episodes reported in the table is 207, because the same aggressor acted upon more than one healthcare worker; therefore, 207 is the number of healthcare workers who experienced aggressive episodes and reported the shift. The care units that were involved the most are the Psychiatry department (36.2%) and the Emergency Room (33.4%). With respect to the total number, 58% of the events involved 2 to 4 healthcare workers. In the comparison between work shifts, statistically significant differences with a *p*-value < 0.05 were found concerning the following variables: care unit area, type of aggression, and category of aggressor.

Table 3 summarizes the characteristics of the aggressor and the number of healthcare workers involved in different care unit areas (Surgery, Medical, Psychiatric, Emergency, Other). No significant *p*-value was found.

Table 4 and Table 5 present data on the hospital management of aggressive events, divided by care unit area (Table 4) and by gender (Table 5). Table 4 reports a statistically significant difference (*p* < 0.01) between certified psychological outcome and the identification of contributing elements, whereas Table 5 reports no statistically significant differences in the hospital management of aggressive events between males and females.

### 3.2. Join Point Regression Analyses

**Figure 1:**
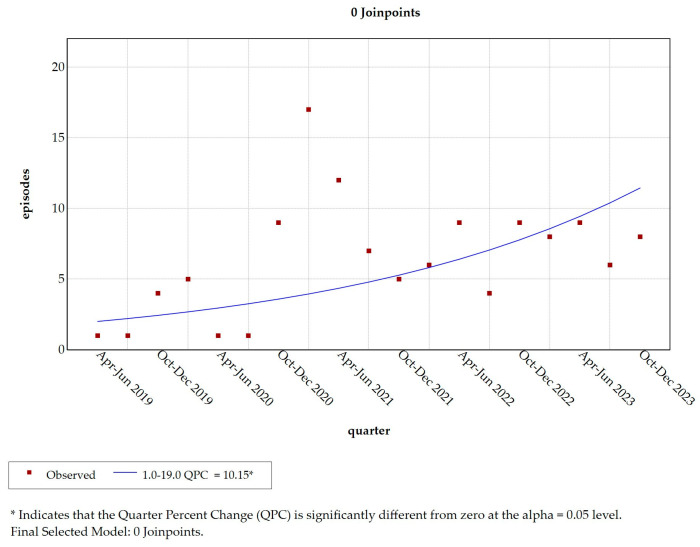
Simple linear join point model (zero join points). Legend: QPC = Quarter Percent Change; * = *p*-value < 0.05.

A simple linear join point model (with zero join points) from April 2019 to December 2023 revealed a significant increase in the reporting of aggression episodes throughout the observation period (QPC = 10.153; t = 3.11; Prob > |t| = 0.006; *p* = 0.022) (Figure 1).

**Figure 2:**
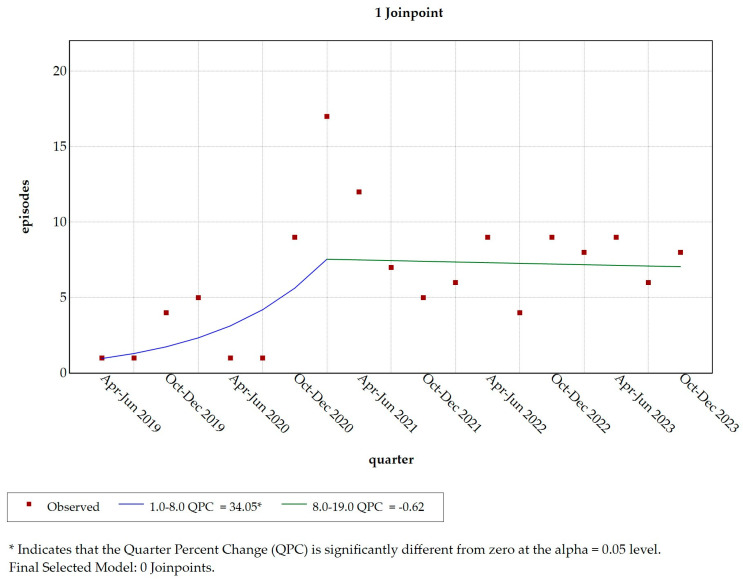
Join point regression analysis with 1 join point. Legend: QPC = Quarter Percent Change; * = *p*-value < 0.05.

By generating a model with one join point, we found a significant increase in the reporting of aggression episodes from April to June 2019 to January to March 2021 (QPC = 34.056; t = 2.47; Prob > |t| = 0.026; p = 0.005). However, no significant change occurred from January to March 2021 to October to December 2023 (QPC = −0.616; t = −2.26; Prob > |t| = 0.040; *p* = 0.765) (Table 6 and Figure 2).

## 4. Discussion

When discussing workplace violence, one concern is that the prevalence varies significantly from one study to another, depending on the sort of violence measured, the employment sector and the country evaluated, and which definition and metrics were utilized [24]. Regardless of the chosen definition of workplace violence [1], and disregarding the frequency of its occurrence, any form of occupational violence should not be acceptable, as personal safety should be a priority in any professional environment, including healthcare settings [13].

Looking at our results, they are consistent with what is being reported in the literature, starting from our findings regarding the higher proportion of female workers than males as victims of aggressiveness and violence [4,13], and concluding with the prevalence of nonphysical violence, with verbal abuse being the most typical form [16,25]. Interestingly enough, our findings confirm that nurses have the highest exposure to violence, followed by physicians and other healthcare professionals [26], but the difference is not statistically significant, even if this could originate from the reduced size of our sample.

In regard to the setting, EDs and mental health settings reported higher levels of violent exposure compared to other settings, and, even if no statistically significant difference was found in our sample, this is confirmed by other studies [16]; this is to be expected, considering that these settings are known to be risky for violence [7,8,25,26]. The aggressor is typically a patient, less often a relative or a friend, as the literature has reported before [8].

In the authors’ opinion, what is particularly relevant is the fact that our RMU activated a psychological service for all the healthcare workers involved, regardless of the type of aggression, as violence is recognized to have detrimental effects on employees’ mental health and general well-being [4,25]; moreover, research on the profound and multiple possible outcomes of workplace violence is still in its infancy [24], making the development and implementation of this kind of service an excellent place to start. Such service was activated as soon as the victim reported the aggression, and, in the majority of the cases, it was followed by a Clinical Risk Management Audit in order to identify contributing and reducing elements; this is a fundamental step in order to recognize the organizational and/or structural changes needed to reduce the risk of a repetition of the same event. In addition, when looking at the hospital’s response to an aggressive event in Table 5, there were no statistically significant differences between males and females, showing that there is no gender bias in the hospital’s handling of the consequences of violence against healthcare workers. This is especially important as, to the authors’ knowledge, the current study is the first of its kind that analyses the hospital’s response to such an event, its protocols, and how they are implemented; therefore, it should prove to serve as a useful point of refence for healthcare executives developing suitable strategies to analyze and reduce workplace violence.

As for the significant increase in the reporting of aggression episodes from April 2019 to December 2023, this is difficult to interpret: for instance, even healthcare workers disagree with one another, especially considering the COVID-19 pandemic, with some reporting an increase in the frequency of violence, others an unchanged frequency, and yet others a drop in frequency [4]. Moreover, seeing as the hospital has been promoting a culture of reporting among healthcare workers since 2018, it is possible that such an increase is connected to the growing awareness rather than to a real increase in violent events; this belief is also enhanced by the creation of the reporting form by the RMU in 2018: before that year, there are no documented cases of violence against healthcare workers, as most of these events are hard to capture electronically, and self-reporting is still the most reliable approach. Furthermore, despite the small size of our sample, it seems that the introduction of the new law in 2020 [19] did not have an impact on the frequency of these events, contrary to our beliefs. However, it is worth mentioning that any reduction in aggressive events registered in the ER since 2022 is to be connected firstly to the introduction of new measures during 2022 and secondly to the overall organizational and structural changes applied in 2023, with the increased presence of law enforcement, greater efficiency in the management of arrivals (especially low priority tags), and a better information sharing system on the status of patients’ health to family members [22].

Lastly, looking at the event classification, during the whole period, there is a greater number of adverse events than of sentinel events, as the former are almost four times the amount of the latter (150 and 41, respectively). This is to be considered positive due to the fact that it highlights how the personnel does not only report the most severe events, which are those that result in a temporary or permanent disability (sentinel events), but they also report the events that resulted in a nonexistent or minor disability (adverse events); this is extremely relevant, as nonphysical forms of violence in healthcare settings are, as a general rule, significantly underreported [8]. Nonetheless, in the literature, it is difficult, if not impossible, to find an exhaustive analysis regarding event classification when the victims of aggression are healthcare workers.

Overall, it is a common point in the literature, and the authors agree, that improved protection measures are needed to create a safe working environment [13], to the point that HWs, informal caregivers, and patients believe that restrictive interventions are needed to manage aggressive events [27], especially when considering the high prevalence of violent acts experienced by healthcare workers [3]. For this reason, and taking into consideration the positive effect on our ER of an in-depth analysis and its subsequent organizational and structural changes [22], it is necessary to involve different stakeholders in various initiatives whose aim is to understand the current situation and to identify common problems in order to, eventually, create new approaches to reduce the impact and the amount of aggressive events [27].

This study has some limitations, which include the reduced sample size and the inclusion of one hospital only; moreover, there is the non-consideration of the level of job experience as a risk factor, and there was no in-depth analysis regarding the psychological consequences of the aggression on the healthcare workers.

## 5. Conclusions

Even if the results are, in some cases, difficult to interpret, it is undeniable that the promotion of a culture of reporting inside a University Hospital of Central Italy led to an established reporting ethic among healthcare workers, as seen by the steadily rising number of nonphysical forms of violence disclosed. However, contrary to the researchers’ expectations, the implementation of the new law in 2020 had no impact on the frequency of aggressive episodes.

Statistically significant differences were found with regard to the type of aggression and category of aggressor; this will enable the hospital management executives to classify and assess risk levels and take appropriate action. Moreover, to the authors’ knowledge, this is the first time that a psychological service for all the healthcare workers involved is immediately activated after the event is reported, and where the hospital’s management of aggressive events is taken into consideration. Particularly relevant, from the researchers’ perspective, is the lack of gender bias in the hospital’s handling of violent incidents.

## Figures and Tables

**Table 1:** Characteristics of the sample, aggressor, and healthcare workers involved. Statistical analysis was applied to find statistically significant differences between males and females.

	All	Females **	Males **	*p*-Value
**n**	209	132	76	-
**%**	100	63	36	-
**Age (year), mean (SD, min–max) ^^^**	43.5 (±9.8, 24–64)	44.5 (±10.7, 24–64)	42.0 (±8.1, 24–60)	**<0.001 °**
**Number of HWs involved**	**n (%)**	**n (%)**	**n (%)**	0.255 ^#^
Single HWs involved	86 (41)	53 (40)	32 (42)	
2–4 HWs involved	120 (58)	76 (58)	44 (58)	
>4 HWs involved	3 (1)	3 (2)	0 (0)	
**Profession**				0.254 *
Nurse	160 (77)	100 (76)	59 (78)	
Doctor	28 (13)	21 (16)	7 (9)	
Other	21 (10)	11 (8)	10 (13)	
**Type of aggression**				**0.042** *
Verbal	107(51)	75 (57)	31 (41)	
Physical	25 (12)	17 (13)	8 (10)	
Mixed	63 (30)	31 (23)	32 (42)	
Not specified	14 (7)	9 (7)	5 (7)	
**Shift involved ^@^**				0.480 *
Morning shift (8–14)	85 (41)	55 (42)	30 (39)	
Afternoon shift (14–20)	72 (34)	47 (36)	24 (32)	
Night shift (20–8)	50 (24)	28 (21)	22 (29)	
**Category of aggressor ^§^**				0.238 *
Patient	150 (72)	90 (68)	60 (79)	
Family member or visitor	49 (23)	34 (26)	14 (18)	
**Outcome of event**				0.108 ^#^
No damage	76 (36)	44 (33)	32 (42)	
Light damage	24 (12)	12 (9)	12 (16)	
Moderate damage	28 (14)	19 (14)	9 (12)	
Severe damage	3 (1)	1 (1)	2 (2)	
Not specified	78 (37)	56 (43)	21 (28)	

Legend: x (y) = number (percentage on the total). *p* < 0.05 are written in bold. * Chi-squared test used. ° T-test used (heteroskedastic with two tails). # Fisher’s exact test used. ** Excluded one person from sample: gender not reported. ^ Age not reported in 24 cases out of 209. @ Shift not reported in 2 cases out of 209. ^§^ Total number of aggressors is 131; the amount reported on the table is equal to the number of HWs (209) because the same aggressor acted upon more than one HW. Category of aggressor was not reported in 10 cases out of 209.

**Table 2:** Comparison of units where aggressive events happened, number of HWs involved, and shifts when the events were sparked.

	All	Morning Shift	Afternoon Shift	Night Shift	*p*-Value
**n**	207	85	72	50	-
**%**	100	41	35	24	-
**Care unit area involved**	**n (%)**	**n (%)**	**n (%)**	**n (%)**	**<0.001 ^#^**
Surgery	13 (6)	1 (1)	12 (17)	0 (0)	
Medical	44 (21)	26 (31)	7 (10)	11 (22)	
Psychiatric	75 (36)	34 (40)	24 (33)	17 (34)	
Emergency	69 (34)	19 (22)	28 (39)	22 (44)	
Other	6 (3)	5 (6)	1 (1)	0 (0)	
**Number of HWs involved**					0.814 ^#^
Single HWs involved	84 (41)	37 (44)	31 (43)	16 (32)	
2–4 HWs involved	120 (58)	46 (54)	40 (56)	34 (68)	
>4 HWs involved	3 (1)	2 (2)	1 (1)	0 (0)	
**Profession**					0.225 ^#^
Nurse	159 (77)	60 (71)	55 (76)	44 (88)	
Doctor	27 (13)	13 (15)	10 (14)	4 (8)	
Other	21 (10)	12 (14)	7 (10)	2 (4)	
**Type of aggression ^@^**					**0.031** *
Verbal	106 (51)	51 (60)	41 (57)	14 (28)	
Physical	25 (12)	9 (11)	9 (12)	7 (14)	
Mixed	62 (30)	23 (27)	18 (25)	21 (42)	
**Category of aggressor ^§^**					**<0.001 ^#^**
Patient	149 (72)	62 (73)	41 (57)	46 (92)	
Family member or visitor	48 (23)	15 (18)	29 (40)	4 (8)	
**Outcome of event**					0.138 ^#^
No damage	76 (37)	31 (37)	20 (28)	25 (50)	
Light damage	24 (12)	8 (9)	8 (11)	8 (16)	
Moderate damage	27 (13)	13 (15)	9 (13)	5 (10)	
Severe damage	3 (1)	1 (1)	1 (1)	1 (2)	
Not specified	77 (37)	32 (38)	34 (47)	11 (22)	

Legend: x (y) = number (percentage on the total). *p* < 0.05 are written in bold. * Chi-squared test used. # Fisher’s exact test used. @ Category of aggressor not reported in 14 cases out of 207. ^§^ Total number of aggressors is 131; the amount reported on the table is equal to the number of HWs from whom the shift was reported (207) because the same aggressor acted upon more than one HW. Category of aggressor was not reported in 10 cases out of 207.

**Table 3:** Characteristics of aggressors. Statistical analysis was applied to find statistically significant differences between five care unit areas.

	All	Surgery	Medical	Psychiatric	Emergency	Other	*p*-Value
**n**	131	8	26	43	48	6	-
**%**	100	6	20	33	37	4	-
**Number of HWs involved**	**n (%)**	**n (%)**	**n (%)**	**n (%)**	**n (%)**	**n (%)**	0.874 ^#^
Single HWs involved	87 (67)	6 (75)	16 (61)	26 (61)	34 (71)	5 (83)	
2–4 HWs involved	41 (31)	2 (25)	8 (31)	16 (37)	14 (29)	1 (17)	
>4 HWs involved	3 (2)	0 (0)	2 (8)	1 (2)	0 (0)	0 (0)	
**Category of aggressor ^@^**							0.237 ^#^
Patient	93 (71)	1 (13)	11 (42)	43 (100)	36 (75)	2 (33)	
Family member or visitor	30 (23)	5 (63)	13 (50)	0 (0)	10 (21)	2 (33)	

Legend: x (y) = number (percentage on the total). # Fisher’s exact test used. @ Category of aggressor not reported in 8 cases out of 131.

**Table 4:** Hospital management of aggressive events. Statistical analysis was applied to find statistically significant differences between five care unit areas.

	All	Surgery	Medical	Psychiatric	Emergency	Other	*p*-Value
**n**	209	13	45	75	70	6	-
**%**	100	6	22	36	33	3	-
**Event classification ^@^**	**n (%)**	**n (%)**	**n (%)**	**n (%)**	**n (%)**	**n (%)**	0.155 ^#^
Adverse event	150 (72)	6 (46)	35 (78)	53 (71)	52 (74)	4 (67)	
Sentinel event	41 (20)	0 (0)	8 (18)	22 (29)	9 (13)	2 (33)	
**Clinical Risk Management Audit ^^^**							0.250 ^#^
Yes	178 (85)	13 (100)	28 (62)	67 (89)	66 (94)	4 (67)	
No	6 (3)	0 (0)	1 (2)	1 (1)	3 (4)	1 (17)	
**Certified psychological outcome ^$^**							**0.011 ^#^**
Yes	24 (11)	0 (0)	1 (2)	11 (15)	11 (16)	1 (17)	
No	117 (56)	11 (85)	34 (76)	33 (44)	36 (51)	3 (50)	
**Identification of contributing elements**							**<0.001 ^#^**
Yes	161 (77)	10 (77)	21 (47)	66 (88)	61 (87)	3 (50)	
No	48 (23)	3 (23)	24 (53)	9 (12)	9 (13)	3 (50)	
**Identification of reducible elements**							0.653 ^#^
Yes	142 (68)	7 (54)	31 (69)	52 (69)	49 (70)	3 (50)	
No	67 (32)	6 (46)	14 (31)	23 (31)	21 (30)	3 (50)	

Legend: x (y) = number (percentage on the total). *p* < 0.05 are written in bold. # Fisher’s exact test used. @ Event classification not reported in 18 cases out of 209. ^ Clinical Risk Management Audit not reported in 25 cases out of 209. $ Psychological outcome not reported in 68 cases out of 209.

**Table 5:** Hospital management of aggressive events between males and females.

	All	Females **	Males **	*p*-Value
**n**	209	132	76	-
**%**	100	63	36	-
**Event classification ^@^**	**n (%)**	**n (%)**	**n (%)**	0.281 *
Adverse event	150 (72)	96 (73)	53 (70)	
Sentinel event	41 (20)	22 (17)	19 (25)	
**Clinical Risk Management Audit ^^^**				0.670 ^#^
Yes	178 (85)	113 (86)	64 (84)	
No	6 (3)	3 (2)	3 (4)	
**Certified psychological outcome ^$^**				0.168 *
Yes	24 (12)	12 (9)	12 (16)	
No	117 (56)	76 (58)	41 (54)	
**Identification of contributing elements**				0.151 *
Yes	161 (77)	98 (74)	63 (83)	
No	48 (23)	34 (26)	13 (17)	
**Identification of reducible elements**				0.114 *
Yes	142 (68)	85 (64)	57 (75)	
No	67 (32)	47 (36)	19 (25)	

Legend: x (y) = number (percentage on the total). * Chi-squared test used. # Fisher’s exact test used. ** Excluded one person from sample: gender not reported. @ Event classification not reported in 18 cases out of 209. ^ Clinical Risk Management Audit not reported in 25 cases out of 209. $ Psychological outcome not reported in 68 cases out of 209.

**Table 6:** Summary of significant “join point regression analysis” models. Legend: QPC = Quarter Percent Change.

Segment	Lower Endpoint	Upper Endpoint	QPC	Lower CI	Upper CI	*p*-Value
1	Apr–Jun 2019	Oct–Dec 2023	10.153	1.345	19.597	**0.022**
**Segment**	**Lower Endpoint**	**Upper Endpoint**	**QPC**	**Lower CI**	**Upper CI**	***p*-Value**
1	Apr–Jun 2019	Jan–Mar 2021	34.046	11.242	235.921	**0.005**
2	Jan–Mar 2021	Oct–Dec 2023	−0.616	−52.723	10.245	0.765

Legend: *p* < 0.05 are written in bold.

## Data Availability

The raw data supporting the conclusions of this article will be made available by the authors on request.

## Abbreviations

The following abbreviations are used in this manuscript:

CRRC	Centro Regionale Rischio Clinico—Regional Center of Clinical Risk
CDC	Centers for Disease Control and Prevention
ED	Emergency Department
ER	Emergency Room
HW	healthcare worker
ILO	International Labour Organization
RMU	Risk Management Unit
QPC	Quarter Percent Change
WHO	World Health Organization
